# In Vivo Assessment of Osseous Wound Healing Using a Novel Bone Putty Containing Lidocaine in the Surgical Management of Tooth Extractions

**DOI:** 10.1155/2012/894815

**Published:** 2012-06-13

**Authors:** Akshay Kumarswamy, Antonio Moretti, David Paquette, Ricardo Padilla, Eric Everett, Salvador Nares

**Affiliations:** ^1^Department of Periodontology, School of Dentistry, The University of North Carolina at Chapel Hill, Manning Drive and Columbia Street, Campus Box 7450, Chapel Hill, NC 27599-7450, USA; ^2^Department of Periodontics, College of Dental Medicine Nova Southeastern University, 3301 College Avenue, Fort Lauderdale-Davie, FL 33314-7796, USA; ^3^School of Dental Medicine, Stony Brook University, 150 Rockland Hall, Stony Brook, NY 11794-8700, USA; ^4^Department of Diagnostic Sciences, School of Dentistry, The University of North Carolina at Chapel Hill, Manning Drive and Columbia Street, Campus Box 7450, Chapel Hill, NC 27599-7450, USA; ^5^Department of Pediatric Dentistry, School of Dentistry, The University of North Carolina at Chapel Hill, Manning Drive and Columbia Street, Campus Box 7450, Chapel Hill, NC 27599-7450, USA

## Abstract

*Objective*. This preclinical pilot study evaluated the systemic, radiographic, and histological responses to bone putty containing lidocaine in a canine tooth extraction model. *Methods*. In five beagle dogs the right mandibular premolars were extracted and sockets grafted with (1) xenograft particulate bone and a collagen sponge plug (control), (2) bone putty alone, (3) bone putty mixed with xenograft (3 : 1), or (4) xenograft sandwiched between bone putty. At 6 weeks post-op, the systemic and local responses were evaluated using a blood chemistry panel, micro-CT, and histological analyses. *Results*. No significant differences in blood chemistries were noted at 6 weeks postgrafting compared to baseline. Sockets grafted with either bone putty formulation demonstrated comparable radiographic and histologic evidence of bone healing compared to control sockets. *Conclusions*. Our preclinical results indicate that this bone putty appears to be a safe biocompatible device that may be useful in the postoperative management of tooth extractions.

## 1. Introduction

Exodontia is a common dental procedure to address severely decayed, periodontally involved, nonrestorable, or impacted teeth. These procedures are typically performed under local anesthesia but unfortunately can be associated with significant postoperative pain after alleviation of the local anesthetic agent, particularly when 3rd impacted or partially impacted molars are extracted [1, 2]. Further, prolonged bleeding can be a problem especially in patients on anticoagulant therapy [3]. To address post-operative discomfort, analgesics, including narcotics, are often prescribed. These analgesics tend to have 4–6 hours duration of action when taken orally, and may require multiple dosing of up to 4–6 tablets daily [4]. Unwanted side effects of these agents are frequent and have been linked to the gastrointestinal, cardiovascular, and renal systems [5].

Prolonging the duration of analgesia without increasing systemic levels of analgesic agents becomes a desirable goal in clinical practice. Local drug delivery systems have been developed in dentistry but very few addressed delivery of anesthetic or analgesic agents. In fact, an online search of the MEDLINE database using key words “dental,” “local,” “delivery,” “anesthetic,” and “analgesic” identified only 1 report [6] thus highlighting the need for further study. Xybrex bone putty (Orthocon, Inc., Irvington, NY, USA) is an implantable, hand-moldable (with a consistency similar to modeling clay), and absorbable delivery system that delivers a controlled release of lidocaine. Its constituents are a bioresorbable mixture of fatty acid salt (a wax-like tamponade), 16% (w/w) lidocaine, liquid vitamin E (for handling properties), and alkylene oxide copolymer (a dispersing agent). It has been previously shown to provide several days of functional sciatic nerve blockade in rats [7] and is currently being marketed to orthopedic surgeons for local pain management and bone hemostasis. It is possible that hemostatic bone putty containing a slow release anesthetic may be therapeutically beneficial in the post-operative management of simple and complicated exodontia. Further, a mixture of particulate bone grafting material and bone putty may provide the added benefit of helping conserve the three-dimensional architecture of the extraction socket in sites designated for dental implant placement [8].

The main objectives of this preclinical pilot study were to assess the safety profiles, biocompatibility, and healing responses of oral tissues to this bone putty in a canine tooth extraction model.

## 2. Materials and Methods

### 2.1. Animal Care

The animal experimental protocol was reviewed and approved by the Institutional Animal Care and Use Committee (IACUC) of The University of North Carolina at Chapel Hill. Five female beagle dogs approximately 1 year old and weighing approximately 10 kg each were used in this study. All dogs had a full set of erupted permanent teeth that were free of pathology.

### 2.2. Surgical Procedure

The canine extraction site model was used for this study [9]. The animals were anesthetized intravenously with 4–6 mg/kg sodium pentothal. Once under anesthesia, the animals were intubated to maintain a patent airway. The level of anesthesia was maintained and monitored with the help of a blood pressure cuff and pulse oximeter by a veterinarian. Thereafter, 2% lidocaine was infiltrated into the buccal and lingual tissues of the right side of the mandibular arch. The mandibular right 1st, 2nd, 3rd, and 4th premolars were hemisected with a thin fissure bur under copious irrigation and atraumatically extracted with elevators and forceps. The extraction sockets were thoroughly debrided and grafted with one of the four treatment arms: (a) bovine xenograft particulate (BioOss, Osteohealth Inc., Shirley, NY, USA) and collagen plug (CollaPlug-Zimmer Dental, Carlsbad, CA, USA) (control socket (X)), (b) bone putty only (Orthocon, Inc.) (Bp), (c) xenograft particles sandwiched between layers of bone putty placed in the coronal and apical aspect (Bp-X-Bp), (d) a 3 : 1 mixture by weight of bone putty and xenograft particles, respectively (3Bp-X). This mixture was chosen based on preliminary studies (data not shown). A staggered grafting approach was used between animals and sockets and all were sutured and stabilized using 4-0 chromic gut suture.

### 2.3. Postoperative Care and Euthanasia

The dogs were maintained on a soft diet for the entire duration of the study. Post-operatively, ibuprofen 400 mg and amoxicillin 500 mg were given orally three times a day for 3 and 7 days, respectively. Visual observations of soft tissue healing were made every two days for 6 weeks. After 6 weeks, the animals were euthanized with an overdose of pentobarbital (120 mg/kg IV) through the carotid arteries. A mixture of 5% glutaraldehyde and 4% formaldehyde was infused and mandibles dissected *en bloc*. The mandibles were further resected from the canine to the 1st molar with a high speed disc bur and placed in 4% paraformaldehyde.

### 2.4. Blood Chemistry Panel

Blood was drawn from each animal preoperatively and at 6 weeks post-operatively and assayed by chemistry panel inclusive of liver function tests using the Superchem Canine Profile Test (Antech Diagnostics, Lake Success, NY, USA).

### 2.5. Radiographic Analysis

Conventional 2-dimensional radiographs of the mandibular hemisections were taken immediately after resection (Faxitron X-ray Lincolnshire, IL, USA). This was followed by microcomputed tomography (*μ*CT) scan (SCANCO Medical *μ*CT 40, Brüttisellen, Switzerland) to acquire basis images from which axial slices and cross-sectional images were generated. Each slice was 0.07 mm in the buccolingual direction with 1144 slices obtained per hemisected mandible at a resolution of 1024 × 1024 pixels. Each slice was then examined at 2x magnification and the sockets marked as the region of interest. All material present within the region of interest in each socket was included and determined to be the “total volume” (TV). After scanning, the 3D datasets were segmented by using individual global thresholds, above which all pixels are considered bone, and below which all pixels are considered nonbone. The threshold of each dataset was determined using an adaptive method, where the gray level data set is segmented at different levels. The threshold, where the volume fraction changes the least, that is, the steepest gradient of gray levels, was chosen as the threshold for the data set according to manufacturer's recommendations (SCANCO Medical). These *μ*CT images were then analyzed in 3D using *μ*CT analysis software (SCANCO Medical) according to manufacturer's instructions. Ratio of bone volume (BV) to TV of extraction socket and mean bone mineral density (BMD) measurements (as measured with mg HA/mm^3^) were obtained. To obtain baseline density measurements, the materials were placed into sterile 1.5 mL Eppendorf tubes in identical configurations to the grafted sockets and scanned by *μ*CT as described above.

### 2.6. Histological Analysis

After *μ*CT imaging, individual bone blocks containing the entire socket were obtained by sectioning in the mesial-distal direction. Prior to sectioning, the full extent of each socket was mapped radiographically using 22 gauge wire wrapped around the interbony aspect of each socket. Thereafter, the blocks containing the individual sockets were decalcified (Immunocal-Decal Chemical Co., Tallman, NY, USA) and embedded in paraffin. Sections were cut in the buccolingual dimension and stained with hematoxylin-eosin and Gomori trichrome stain. Serial sections were observed under a light microscope (Olympus DX41, Center Valley, PA, USA) using 100x, 200x, and 400x magnification.

### 2.7. Statistical Analysis

A one-way analysis of variance, Kruskal-Wallis Test, and the post-hoc Dunn's multiple comparison tests were used to compare results within the different treatment arms (GraphPad Software Inc., La Jolla, CA, USA). A *P* value <0.05 was considered significant.

## 3. Results

**Figure 1:**
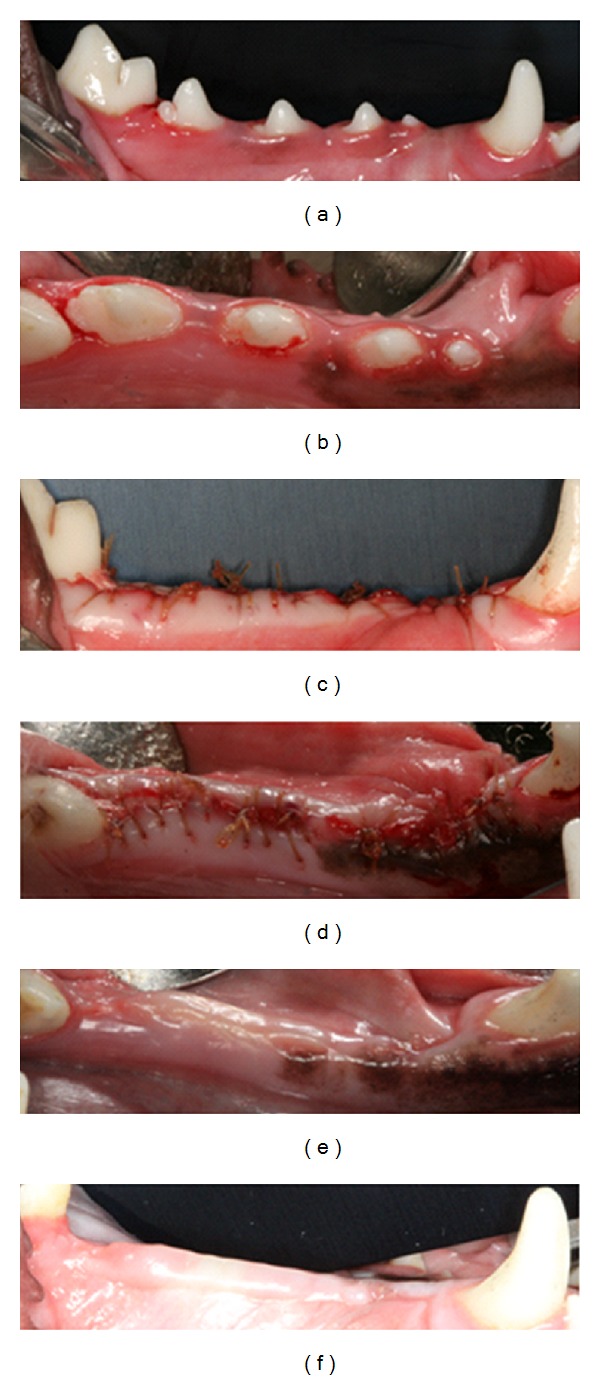
Representative images of preextraction (a) and (b), immediate postextraction (c) and (d), and six-week postextraction healing (e) and (f). Complete closure of extraction sockets and lack of clinical signs of inflammation are evident.

All animals tolerated the procedure well and did not display any overt signs of stress such as elevated body temperature, altered behavior, or weight loss throughout the entire duration of the study. We noted immediate hemostasis when the putty was applied into the extraction socket. The materials were easily applied and remained in the socket throughout the procedure. Further, visual examinations performed 3 times per week over the course of 6 weeks revealed the normal stages of healing of all extraction sockets with no visible evidence of adverse healing events. Preoperative, postextraction, and 6 week post-operative images from a representative dog are shown in Figures 1(a)–1(f). Complete soft tissue closure was noted over each of the extraction sockets in all the animals regardless of the treatment arm. Comparison of pre-operative and 6-week post-operative clinical chemistries revealed chemistry and liver function values that were within the normal reference ranges with no significant differences between groups.

**Figure 2:**
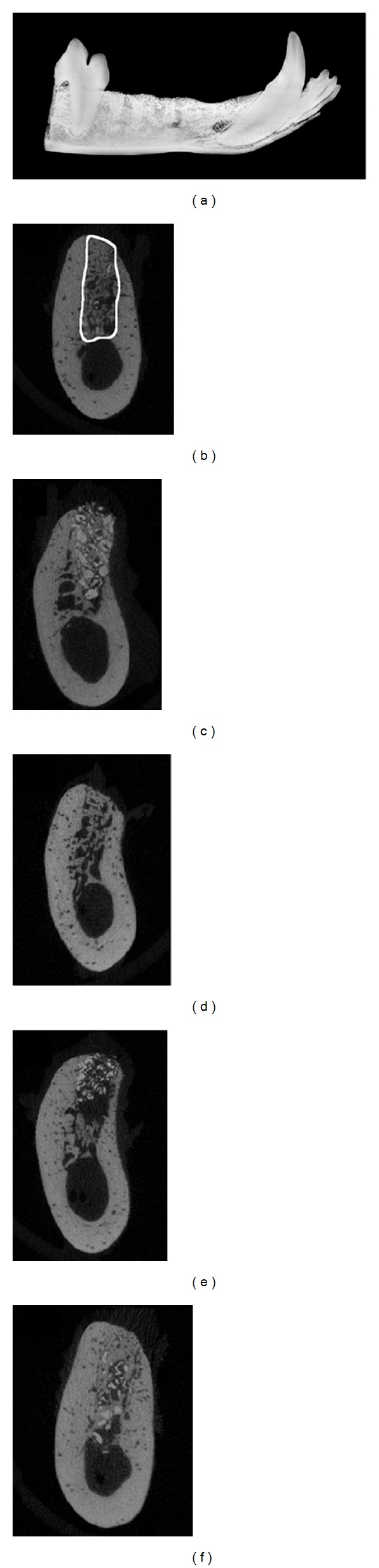
Representative image of a conventional mandibular radiograph (a) and *μ*CT slices (b–f) within the middle of the distal root, 4th premolar extraction sockets after the six-week healing time. (b) *μ*CT image of an extraction socket showing the region of interest analyzed for BV/TV and BMD. (c) Bovine xenograft particulate and collagen plug (X), (d) bone putty only (Bp), (e) xenograft particles sandwiched between layers of bone putty placed in the coronal and apical aspect, (Bp-X-Bp), and (f) a 3 : 1 mixture by weight of bone putty and xenograft particles, respectively (3Bp-X). Residual xenograft particles (appearing denser than the surround bone) can be seen in within the extraction sockets in panels (c), (e), and (f).

**Figure 3:**
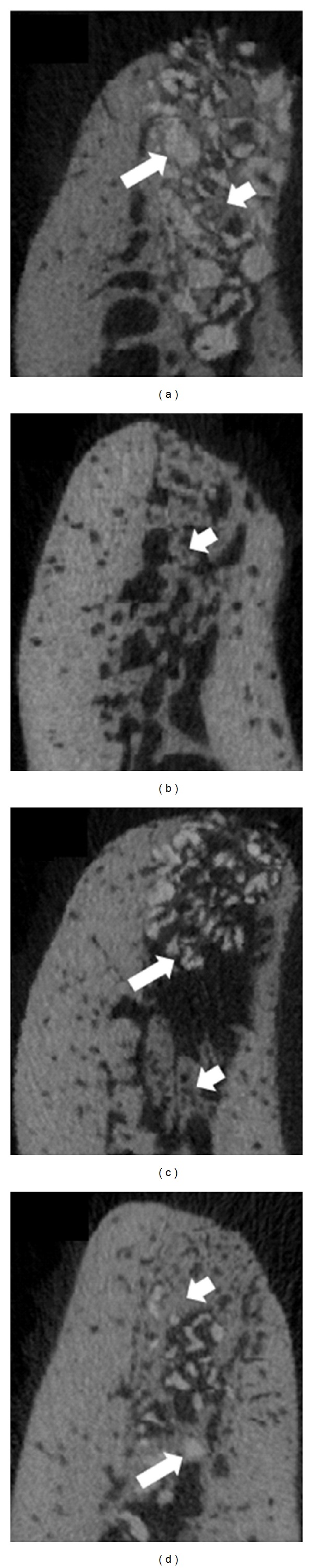
Enlarged representative images of *μ*CT slices within the middle of the distal root, 4th premolar extraction sockets after the six-week healing time. (a) Bovine xenograft particulate and collagen plug (X), (b) bone putty only (Bp), (c) xenograft particles sandwiched between layers of bone putty placed in the coronal and apical aspect (Bp-X-Bp), and (d) a 3 : 1 mixture by weight of bone putty and xenograft particles, respectively (3Bp-X). Residual xenograft particles (appearing denser than the surround bone) can be seen in within the extraction sockets in panels (a), (c), and (d) (long arrows) interspersed between newly formed bone (short arrows, (a)–(d)).

Baseline grafting material density values are noted in Table 1. As expected, the X group recorded the highest density value while the Bp group had the lowest value. The Bp-X-Bp and the 3Bp-X groups recorded density values between those of X and Bp. Representative radiographic imaging of extraction sockets after 6-weeks healing time is shown in Figure 2. Conventional 2D radiographs revealed visually comparable radiographic densities across all sockets (Figure 2(a)). By *μ*CT imaging xenograft particles were visible within the extraction sockets when included in the grafting configuration (Figures 2(c), 2(e), and 2(f)). The particles are clearly evident in enlarged *μ*CT images (long arrows, Figures 3(a), 3(c), and 3(d)). Radiographic evidence of bone formation was also noted within all sockets regardless of grafting configuration (Figures 2(c)–2(f)). New bone formation was clearly evident in enlarged images (short arrows, Figures 3(a)–3(d)). In total, 1144 *μ*CT slices each 0.07 mm thick were taken of each hemisected mandible spanning across the 4 extraction sites. Prior to analysis, regions of interest were identified for each socket on every reconstruction slice (Figure 2(b)), which were used to determine the BV/TV ratios and to calculate BMD. The mean ratio of BV/TV ranged from 0.5578 (55%) to 0.4823 (48%), while the BMD ranged from 841.7 to 816 mm HA/mm^3^ (Table 2). No significant differences were noted between groups (*P* > 0.05). Of note, images from sockets grafted in the BP-X-Bp configuration suggest slower healing rates of bone formation compared to the other groups although the differences in BV/TV and BMD were not statistically different (Figure 2(e)).

**Figure 4:**
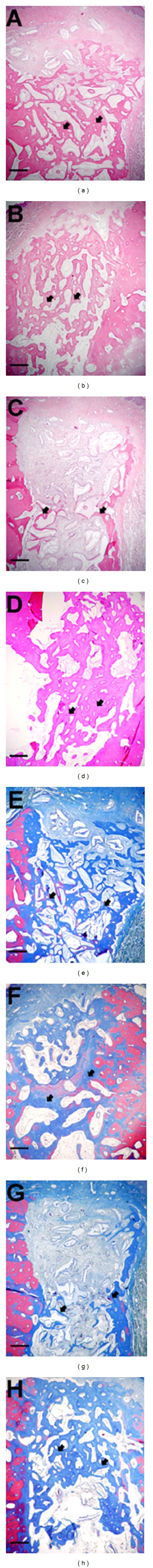
Representative hematoxylin-eosin ((a)–(d)) and Gomori trichrome stain ((e)–(h)) histological images of extraction sockets from the 4th premolar after six weeks of healing time. (a) and (e), bovine xenograft particulate and collagen plug (X), (b) and (f), bone putty only (Bp), (c) and (g), xenograft particles sandwiched between layers of bone putty placed in the coronal and apical aspect, (Bp-X-Bp), and (d) and (h), a 3 : 1 mixture by weight of bone putty and xenograft particles, respectively (3Bp-X). Arrows highlight newly formed bone between xenograft particles or in Bp only grafted sockets (b) and (f). Scale bar = 400 microns, 20x magnification.

**Figure 5:**
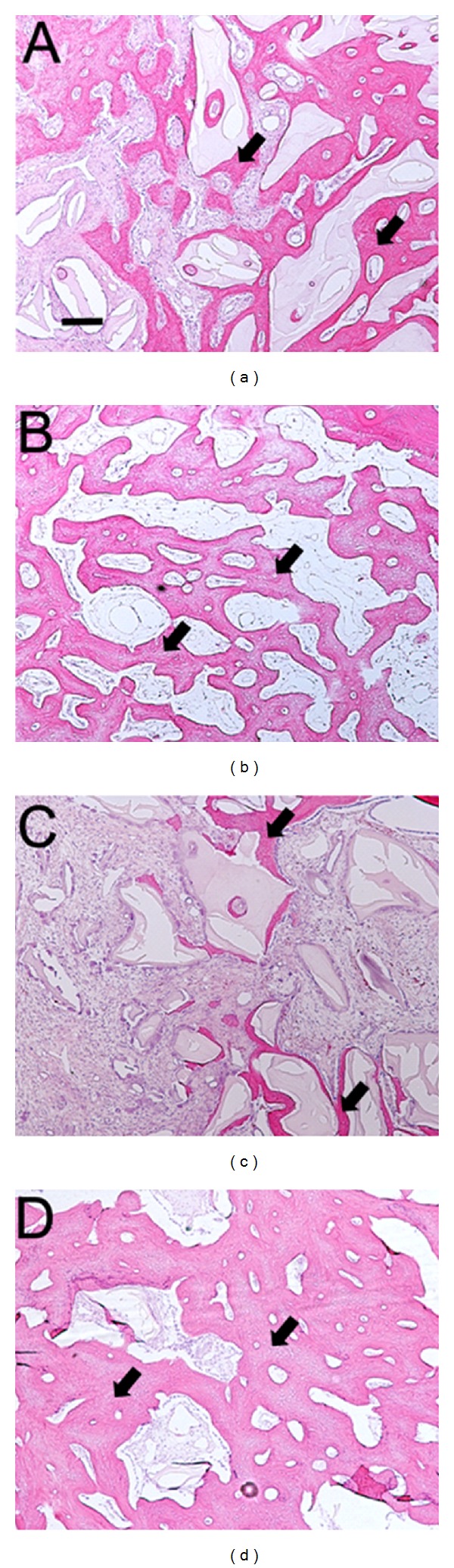
Representative hematoxylin-eosin (a)–(d) images of midsockets from the 4th premolar at six weeks following extractions and material placements. (a) bovine xenograft particulate and collagen plug (X), (b) bone putty only (Bp), (c) xenograft particles sandwiched between layers of bone putty placed in the coronal and apical aspect, (Bp-X-Bp), and (d) a 3 : 1 mixture by weight of bone putty and xenograft particles, respectively (3Bp-X). Arrows highlight newly formed bone between xenograft particles or in Bp only grafted sockets (b). Scale bar = 200 microns, 40x magnification.

Under light microscopy, visual evidence of bone deposition in the form of woven bone was noted within the sockets. As expected, the control sockets grafted with xenograft and collagen sponge (X) contained remnants of xenograft particles between the newly formed bone (arrows, Figures 4(a) and 5(a)). Gomori trichrome staining revealed particulate bone surrounded by osteoid and bone matrix (arrows, Figure 4(e)). Blue staining osteons indicating the presence of maturing bone matrix were intermixed with areas staining a pinkish hue which are suggestive of mature bone. Similarly, the sockets grafted with Bp contained appreciable amounts of vital bone (arrows, Figures 4(b) and 5(b)). Gomori trichrome staining also distinguished new bone and osteoid from native lamellar bone in this group (Figure 4(f)). The healing pattern of the 3Bp-X sites was similar to those of the Bp and X groups with islands of newly formed bone as well as remnants of xenograft particles interposed between the immature bone (arrows, Figures 4(d), 4(h), and 5(d)). However, sockets grafted in the Bp-X-Bp configuration did not appear to contain as much immature/mature bone in the center of the socket compared to the other 3 configurations (arrows, Figures 4(c), 4(g), and 5(c)). This finding was in agreement with the *μ*CT survey. 

## 4. Discussion

The present animal study evaluated the biological responses of oral tissues to a bone putty material containing lidocaine in a tooth extraction model. The main goal was to evaluate the material's safety profiles using both systemic and oral assessments. This was performed using a blood chemistry panel comparing samples collected at baseline and at 6 weeks after extraction/grafting. We also sought to characterize the radiographic and histological responses of the healing extraction sockets.

It has been reported that the adverse effects of local anesthetic agents include but are not limited to paresthesia, ocular complications, allergies, local tissue toxicity, and methemoglobinemia [1]. These agents also affect the cardiovascular and nervous system. The absorbable bone putty used in this study delivers a burst-release of lidocaine. Instead, it delivers a controlled slow-release of the anesthetic agent into the surrounding tissues capable of prolonged analgesia, a desirable post-operative clinical goal in exodontia. Indeed, Wang and colleagues reported that this putty formulation was capable of prolonged and reversible analgesia that lasted at least 10-times longer than lidocaine solutions used for single-injection rat sciatic nerve block without apparent significant local neurotoxicity or systemic toxicity [7]. Nevertheless there is a possibility of lidocaine toxicity both locally and systemically when placed in multiple extraction sockets. In dogs, the total toxic dose of lidocaine ranges from 4.6–19.4 mg/kg [10]. The dogs used in this study had an average weight of about 10 kgs so that the toxic lidocaine dose would need to approach 46 mg to 194 mg. Although we did not measure the blood lidocaine levels immediately after implantation, our clinical, laboratory, radiographic, and histologic data do not suggest systemic or local toxicity. Further, no animal displayed any overt signs of stress such as elevated body temperature, altered behavior or weight loss, and all extraction sockets healed normally. We noted that the blood chemistry values remained within the normal reference range at 6 weeks compared to baseline. Indeed, lidocaine is metabolized mainly in the liver, and the blood chemistry panel did not detect any signs of toxicity, including liver toxicity, in any animal. Levels of albumin, alanine transaminase (SGPT), and aspartate transaminase (SGOT) were within the normal reference range at baseline and 6 weeks.

 Araujo and coworkers reported that marked histological and dimensional changes occur within the first 8 weeks after extraction of mandibular molars in dogs [11]. Thus, by evaluating the biological responses at 6 weeks, local bone metabolism in the presence of Bp alone or when mixed with xenograft particulate bone material can be assessed. Further, a comparison was made to a xenograft and collagen plug formulation which is used clinically for site preservation procedures [12, 13]. Prior to initiation of the study, we compared the handling characteristics of Bp when mixed with xenograft particulate bone at 1 : 1–5 : 1 w/w Bp to bone. We reasoned that particulate bone would provide the added benefit of aiding to conserve the three-dimensional architecture of the extraction socket in sites designated for dental implant placement [8]. The particulate bone is radiographically evident in sockets grafted using the 3Bp-X configuration (Figures 2(f) and 3(d)). Immediate and sustained hemostasis (i.e., bleeding did not last more than a few seconds) in sockets grafted with Bp, Bp-X-Bp, or 3Bp-X was also noted as was complete soft tissue closure at rates comparable to control sockets.

By *μ*CT analysis, the mean BV/TV ratios ranged from approximately 48% to 55% with no statistically significant differences between treatment arms. As mentioned above, this is a period of active bone metabolism in extraction sockets in dogs [11], and we noted evidence of bone formation in all groups (Figures 4(a)–4(h) and 5(a)–5(d)). Occasionally, voids in the Bp-containing sockets were evident histologically which were likely occupied by the putty material. The decalcification and histological processing may have contributed to their formation. Alternatively, portions of the graft materials may have been lost prematurely or the material may be in the advanced stages of resorption. Indeed, in orthopedic surgical procedures, the putty is resorbed in approximately 4 weeks time (personal communication, Orthocon, Inc.). While partial loss of graft materials was possible, complete loss was not detected upon 48-hour visual inspections while *μ*CT and histological analysis confirmed the presence of graft materials in all sockets regardless of grafting configuration.

Regarding bone mineral density, baseline values demonstrated that the Bp grafting configuration was lower than the X configuration consistent with the fact that Bp is not a mineralized product while the particulate material consists of inorganic bovine bone matrix. The xenograft particles did not resorb completely over 6 weeks and were clearly visible both radiographically and histologically in agreement with other studies [14–16]. As a result, this likely contributed to higher density values in the X group, after grafting. Despite these observations, *μ*CT analysis failed to demonstrate statistically significant (*P* > 0.05) differences between the treatment arms suggesting that the bone putty did not interfere with bone metabolism and that mineralization proceeded normally. Bone density increases over time due to calcification of bone matrix [17], and appreciable amounts of newly formed bone were noted radiographically and histologically in sockets containing the bone putty material.

The vast majority of the sockets displayed a minimal inflammatory infiltrate histologically suggesting that the materials were well tolerated. However, we noted radiographically and histologically that healing of sockets grafted in the Bp-X-Bp configuration appeared to proceed at a slower rate compared to the other configurations. The reasons for this finding remain unclear but it is possible that a portion of the coronal aspect of the putty may have been lost prematurely despite suturing along the socket. This was evident radiographically by the apparent extrusion of the xenograft particulate bone material in the coronal direction (Figure 2(e)). Thus, this strategy was not as sound or as well tolerated compared to the other grafting configuration. Indeed, in 1 socket in 1 animal grafted in this configuration, histological evidence of a foreign body reaction was noted. No other socket grafted with either particulate bone or bone putty displayed this histological reaction. Currently the material is FDA approved for use in orthopedic procedures and has been noted to demonstrate a minimal inflammatory reaction in other animal models [7]. However, our finding could also be attributed to the fact that in orthopedic medicine, sterile conditions are used, while in the oral cavity a gnotobiotic state is not achievable in dogs.

The potential limitations of this study were the small sample size, lack of randomization and longitudinal blood sampling, and the absence of dynamic histomorphometry. An untreated extraction socket (clot only) would be useful to compare healing in the absence of graft materials. However, it has been previously reported that over the course of 180 days, the healing of an ungrafted extraction socket in dogs involves a series of events including the formation of a coagulum that was replaced by (i) a provisional connective tissue matrix, (ii) woven bone, and (iii) lamellar bone and bone marrow [18]. The period between ~4 and 8 weeks was marked with mineralized bone in agreement with our study. Human clinical trials will be required to assess the analgesic properties of this material.

## 5. Conclusions

Within the limits of this investigation, this pre-clinical pilot study indicates that bone putty containing lidocaine, when used alone or when mixed with xenograft bone particulate, did not induce adverse local, systemic or radiographic responses. The histological and radiographic assessment indicated comparable bone healing rates compared to control sockets when placed alone or when mixed with xenograft in an extraction socket. Further studies are required to confirm these findings as well as to assess its analgesic properties in humans.

## Figures and Tables

**Table 1:** Baseline density values for each grafting configuration.

Graft material	Baseline density values (mg HA/mm^3^)
X	607.45
Bp-X-Bp	452.71
3Bp-X	435.93
Bp	387.66

**Table 2:** A comparison of bone volume to total volume ratios (BV/TV) and bone mineral density (BMD) values across all treatment arms.

Treatment arm	Bp	X	3Bp-X	Bp-X-Bp	*P*-value
	BV/TV ratios				

Means	0.5578	0.5544	0.4914	0.4823	*P* > 0.05
Std. deviation	0.0522	0.05002	0.1142	0.1143	

	BMD (mg HA/mm^3^)				

Means	816	841.7	822	838.6	*P* > 0.05
Std. deviation	12.7	20.97	11.8	3.911	
